# Strategy for dealing with unfamiliar and thick vessels during microvascular decompression: A first case report of hemifacial spasm caused by a persistent primitive trigeminal artery

**DOI:** 10.1097/MD.0000000000036175

**Published:** 2023-11-24

**Authors:** Kanghee Ahn, Yeong Jin Kim, Tae-Young Jung, Kyung-Sub Moon, In-Young Kim, Shin Jung

**Affiliations:** a Department of Neurosurgery, Chonnam National University Research Institute of Medical Science, Chonnam National University Hwasun Hospital and Medical School, Hwasun, South Korea.

**Keywords:** hemifacial spasm, microvascular decompression, persistent primitive trigeminal artery

## Abstract

**Rationale:**

A persistent primitive trigeminal artery (PPTA) is a rare embryonic cerebrovascular anomaly. Hemifacial spasm (HFS) refers to involuntary contractions of facial muscles caused by the compression of blood vessels against the root exit zone of the facial nerve. There have been no reported cases of PPTA causing neurovascular contact and HFS. Microvascular decompression surgery effectively treats HFS, but operating on strong PPTA vessels poses challenges. We aim to introduce a more efficient approach for overcomes these difficulties and facilitates surgery.

**Patient concerns:**

A 44-year-old male patient without any underlying medical conditions presented to our hospital with involuntary movements of the left side of his face accompanied by numbness in the left maxilla (V2 area).

**Diagnosis:**

Brain magnetic resonance imaging and magnetic resonance angiography showed that PPTA was in contact with the left facial nerve.

**Interventions and outcomes:**

Following a retro-sigmoid craniotomy, we attempted to interpose the facial nerve and the PPTA as an offender vessel, but the decompression was not sufficient. However, after transposing the vessel using the proximal Teflon transposition with interposition technique, the strength of the involuntary movements was reduced. Following surgery, there was no more lateral spreading response, and the patient symptoms improved.

**Lessions:**

In cases where the vessel causing HFS is particularly strong and thick, the proximal Teflon transposition with interposition technique for transposition may be advantageous. This method could simplify and enhance the efficacy of microvascular decompression, without compromising the quality of surgical outcomes.

## 1. Introduction

Hemifacial spasm (HFS) is an involuntary tonic-clonic contraction of the facial muscles that occurs when vessels contact the root exit zone (REZ) of the facial nerve in the brainstem.^[1–3]^ It results from abnormal excitation of the facial nerve nucleus or abnormal transmission of nerve fiber communication signals.^[4–6]^ Surgical exploration has found that HFS is caused by the posterior inferior cerebellar artery (PICA), the anterior inferior cerebellar artery (AICA), the vertebro-basilar artery, and other smaller arteries in 47.2%, 45.9%, 17.5%, and 11.7% of cases, respectively.^[7]^

A persistent primitive trigeminal artery (PPTA) is vascular channels that connect the cavernous segments of the developing internal carotid arteries to a pair of longitudinal neural arteries, which later form the basilar artery.^[8]^ A PPTA is the most common primitive carotid-basilar anastomosis, with an incidence of 0.5% to 1.0% observed by cerebrovascular angiography, and is increasingly recognized by incidental findings in computed tomography angiography and magnetic resonance imaging/magnetic resonance angiography.^[9–14]^

Traditional techniques for inserting Teflon patties directly into the REZ may result in surgical failure or unnecessary pressure due to the large diameter and high pressure of thick vessels.^[15]^ In cases of HFS where tension is heavily applied due to the thick vessel, PPTA, simple interposition alone may not alleviate nerve irritation. Many studies have introduced surgical techniques for microvascular decompression (MVD) in patients with thickened vessels.^[3,16–20]^ These techniques are more challenging, require longer surgeries than traditional MVDs, and carry risks.^[15]^

To the best of our knowledge, no other study has reported a PPTA as the cause of HFS. In this report, we present a case of HFS caused by PPTA as offender vessel and describe the surgical technique used to treat it.

## 2. Case presentation

### 2.1. Case illustration

**Figure 1.**
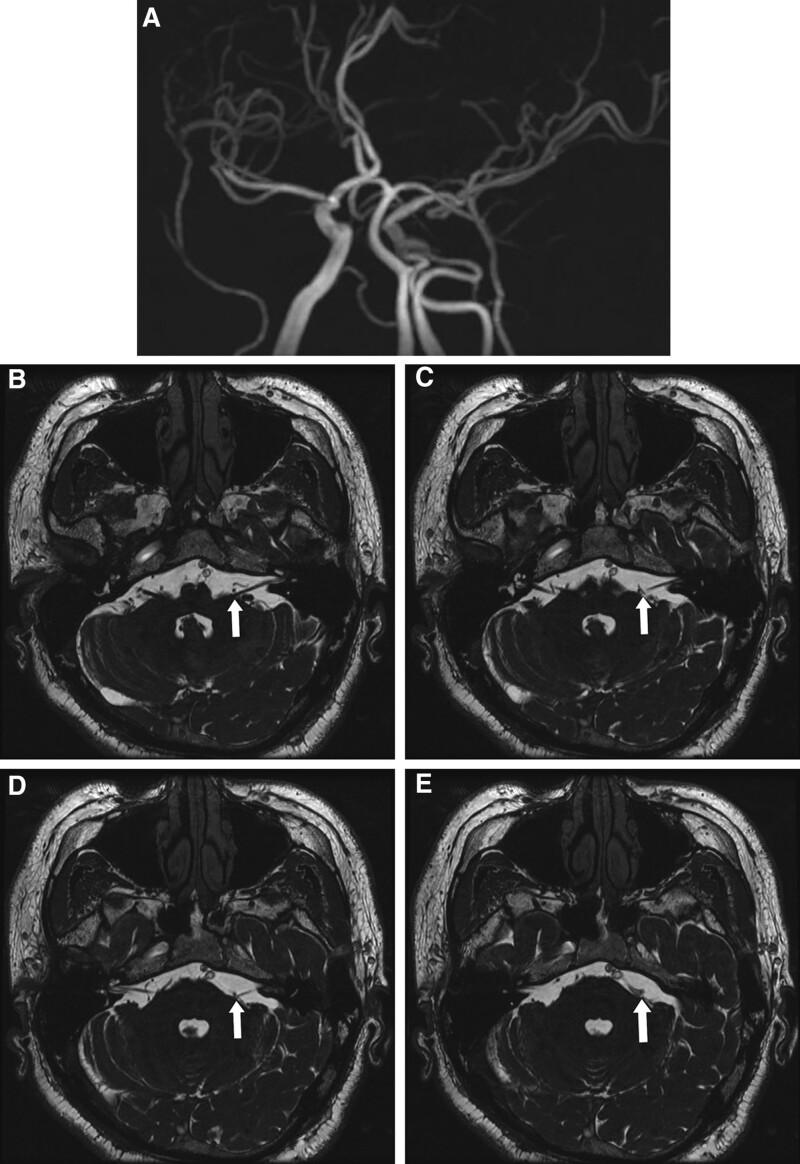
Pre-operative magnetic resonance imaging. (A) Maximum intensity projection magnetic resonance angiography image before surgery. (B–E) Magnetic resonance angiography images showing the neurovascular contact site (white arrows).

**Figure 2.**
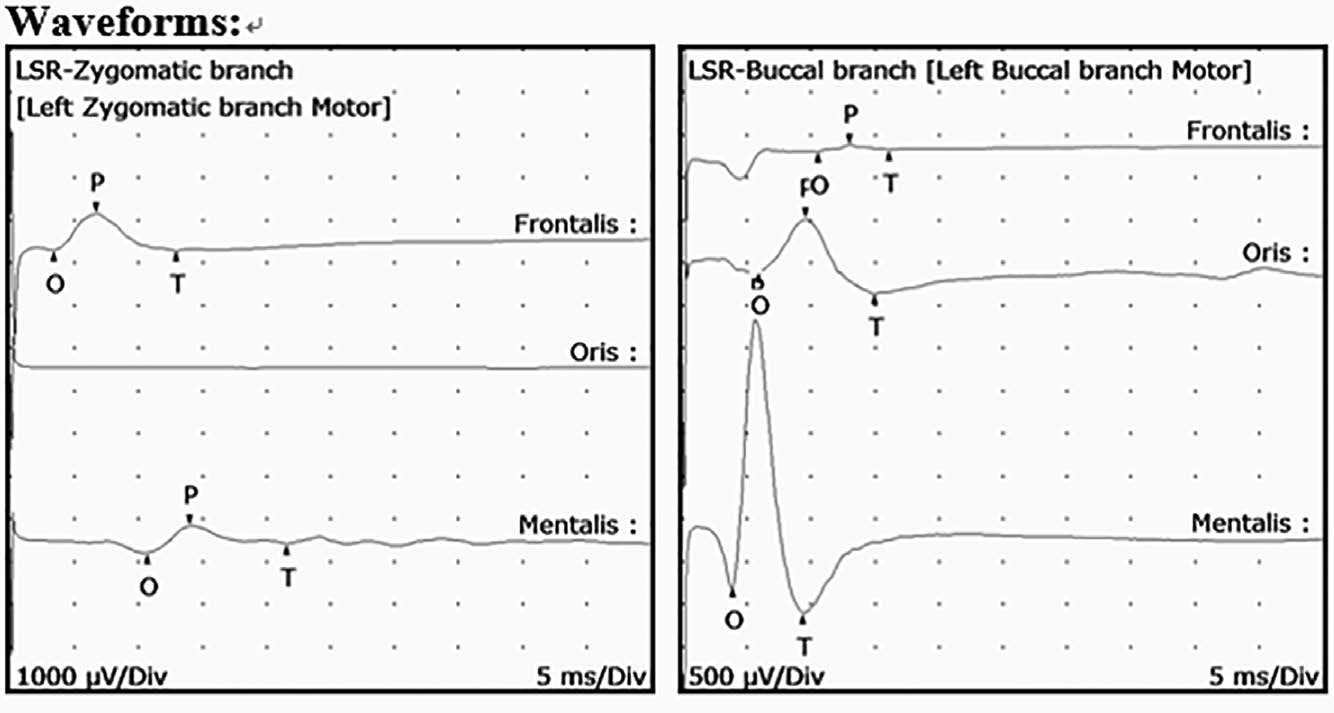
Preoperative lateral spreading responses. (A) EMG recording was conducted on the frontalis and mentalis muscles (left side) in response to stimuli applied on the left zygomatic and buccal branches to evaluate the presence of lateral spreading response. EMG = electromyography.

A 44-year-old male patient with no underlying medical conditions presented to our hospital with involuntary left facial movement and numbness in the left maxillary area (V2) that had persisted for 2 years. Physical examination revealed involuntary facial movement, and brain magnetic resonance imaging and magnetic resonance angiography showed persistent primitive trigeminal arteries on the left side. Fast imaging employing steady-state acquisition showed that the left facial nerve was being compressed from below by the tortuous and elongated left distal PPTA (Fig. 1A–E). Preoperative electromyography (EMG) identified the late spreading response (LSR) on the left zygomatic and buccal branches of the facial nerve (Fig. 2). The patient had previously been diagnosed with left HFS and had received nonsurgical treatment, but his symptoms did not improve with medication. Therefore, MVD was performed.

### 2.2. Intraoperative monitoring

Continuous intraoperative monitoring was performed on the patient using facial EMG and brainstem auditory evoked potentials. EMG monitoring was performed and needle electrodes were inserted intradermally into the frontalis, orbicularis oculi, orbicularis oris, and mentalis muscles to record responses. Stimulating waves (300-μs pulse waves, 5–25 mA) were administered to the zygomatic and buccal branches of the ipsilateral facial nerve, and LSRs were recorded simultaneously at the orbicularis oris and mentalis muscles after stimulation of the zygomatic branch of the facial nerve and at the frontalis and orbicularis oculi muscles after stimulation of the buccal branch of the facial nerve. Muscle-relaxing agents were administered with the induction of anesthesia, and EMG signals were temporarily interrupted.

### 2.3. Surgical procedure

**Figure 3.**
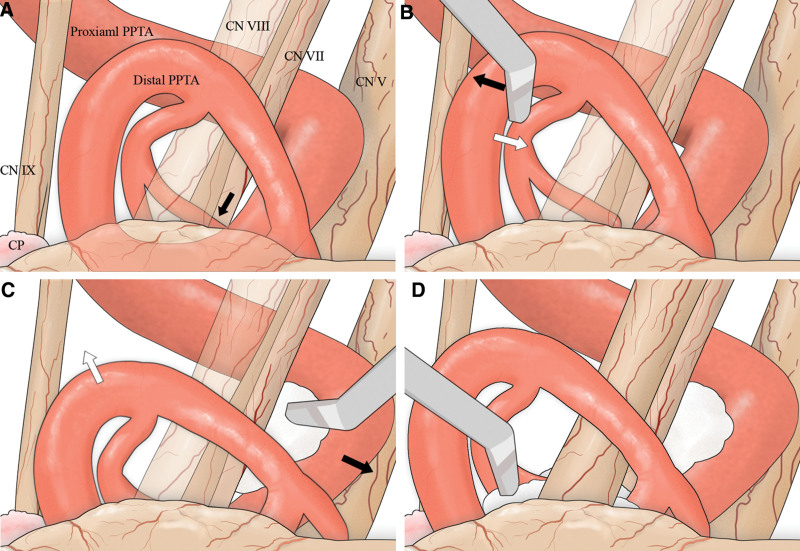
Schematic diagram for proximal Teflon transposition with interposition. (A) The image demonstrates the neurovascular conflict of the left facial nerve caused by distal persistent primitive trigeminal artery (PPTA) (black arrow). (B) Even when pulling the distal PPTA in the opposite direction from the nerve (black arrow), the force (white arrow) pulling it back due to parent vessel tension is substantial. (C) Proximal Teflon transposition with insertion reduced the tension of the parent vessel (black arrow), leading to alleviation of the force exerted on the facial nerve by the distal PPTA (white arrow). (D) Interposition became feasible by inserting Teflon between the distal PPTA and the facial nerve, reducing excessive pressure. CN = cranial nerve, CP = choroid plexus, Prox. = proximal.

A retro-sigmoid suboccipital approach to craniotomy was used, and the skull was removed adjacent to the sigmoid and transverse sinuses (2.0 × 1.0 cm). After dural incision, Cerebrospinal fluid was released by arachnoid trabeculae incision and dissection. After retracting the cerebellar hemisphere and choroid plexus, further arachnoid dissection was performed to identify the REZ of the seventh nerve. The left PPTA was exposed, and the arachnoid completely dissected from the left facial nerve (Fig. 3A). To alleviate the neurovascular conflict, a small piece of Teflon sheet was inserted between the distal PPTA and the pons in the root entry zone (REZ) area. However, this alone was not enough due to the strong tension of the PPTA (Fig. 3B). Therefore, we moved the proximal PPTA to the caudal side and placed a larger Teflon patty between the facial nerve and the proximal PPTA (Fig. 3C). This reduced the tension of the parent vessel and relieved the force exerted on the facial nerve by the distal PPTA. Additionally, we inserted more Teflon patties in the REZ where the facial nerve had contact with the offending vessel. The expanded space created by the PPTA transposition allowed for easier insertion, resulting in lower pressure on the facial nerve (Fig. 3D). This successful interposition effectively reduced the excessive pressure caused by the neurovascular conflict. The patient was monitored by EMG throughout the surgery, and measurements were obtained at following points: initiation of surgery, dural incision followed by cerebrospinal fluid drainage, Teflon insertion, and surgery completion. The LSR disappeared after the Teflon was inserted.

### 2.4. Postoperative outcome

After surgery, the patient did not show any facial movements, and he had no postoperative complications.

## 3. Discussion

HFS is an involuntary tonic-clonic contraction of the facial muscles that occurs when they contact vessels in the REZ of the facial nerve in the brainstem.^[2,3,16]^ It causes abnormal excitation of the facial nerve nucleus or abnormal transmission of nerve fiber communication signals.^[4–6]^ The prevalence of HFS is 11 to 40 per 100,000 people, and the ratio of women to men is 2:1.^[21,22]^ According to other studies, the vessels involved in HFS are the PICA, the AICA, the vertebro-basilar artery, and smaller arteries. Non-surgical treatment of HFS is botulinum injection, which relieves symptoms in about 85% of patients; however, the effect is short-lived, ranging from 3 to 6 months.^[15,23]^

During embryonic development, various anastomoses occur between the carotid artery and the vertebro-basilar system, including the PPTA, hypoglossal artery, otic artery, and prostatic intersegmental artery.^[24]^ These occur during the longest embryonic period and are typically eliminated by the 11.5 to 14 mm embryonic stage.^[25–27]^ In some cases, anastomoses persist into adulthood and remain patent.^[28]^ The trigeminal artery typically degenerates after development of the posterior communicating artery can be maintained continuously for unclear reasons.^[29]^ The PPTA is commonly derived from the walls of the intracavernous internal carotid artery in a posterolateral or posteromedial direction.^[30]^ Within the cavernous sinus, the PPTA is classified as lateral or medial.^[31]^ Both types of PPTA join the distal third of the basilar artery. In some cases, the PPTA, which has no connection with the basilar artery, is directly connected to one of the cerebellar arteries (superior cerebellar artery, AICA, or PICA). This type of PPTA is a PPTA variant.^[32]^ However, in rare cases, there is a link between the PPTA or PPTA variant and cranial neurovascular conflict (trigeminal neuralgia or oculomotor palsy).^[33–35]^ In this case, we found that the PPTA variant caused neurovascular conflict in the facial nerve, and surgery improved the symptoms.

MVD is an effective surgical procedure for patients suffering from HFS. It is effective when blood vessels directly compress the cranial nerves, but if a large, strong blood vessel is compressed, such as the vertebral artery or associated vessels, the outcome of surgery is worse than if a vessel like the PICA or AICA is compressed.^[18,36]^ When such a large vessel is involved, a large vessel transposition is required to facilitate interposition of the neurovascular contact of the REZ. Many effective surgical techniques have been developed, including Teflon sling reposition, dural flap, transposition with a synthetic vascular graft, the double-stick tape technique, the Teflon sling coated with Tisseel (Baxter Healthcare, Deerfield, Illinois, USA) technique, the clip-sling-clip technique, anchoring with a fenestrated clip.^[3,16,18–20,25,36–38]^ However, since these surgical methods are long and complicated, we operated on this patient in a simpler way.^[15]^

To reduce the strength of the PPTA, transposition was implemented, and the proximal Teflon transposition with interposition (PTTI) technique previously developed by our institute was used.^[15]^ This can be done with a mini-craniotomy because it does not require a large field to hang the sling. In cases where Teflon patties are inserted between the REZ and the offender vessel following meticulous arachnoid dissection, interposition may not be sufficient due to the strong tension caused by the PPTA, leading to excessive pressure (Fig. 3A and B). If the Teflon patties are inserted between the proximal PPTA and the facial nerve to perform proximal PPTA transposition, the tension of vessel is reduced to facilitate the interposition (Fig. 3C and D). The pulsatile force is a failure point in other techniques because it causes the transposed vessels to leave their positions in the slings. However, the PTTI technique can reduce the pulsatile force of the proximal vessel. In addition, since the area for fixing the transposed vessels is large, it can be positioned more stably. In our hospital, patients who operated on by this technique showed relatively good prognoses.^[15]^

## 4. Conclusion

This is the first reported case of decompression of a facial nerve using the PTTI technique. We have successfully performed this surgical technique, and we anticipate that it will become more commonly used in the future.

## Author contributions

**Data curation:** Kanghee Ahn, Yeong Jin Kim, Tae-Young Jung, Kyung-Sub Moon, In-Young Kim.

**Resources:** Shin Jung.

**Supervision:** Shin Jung.

**Visualization:** Kanghee Ahn.

**Writing – original draft:** Kanghee Ahn.

**Writing – review & editing:** Shin Jung.
